# Editorial on the Special Issue “The New Frontier of Venous Thromboembolism”

**DOI:** 10.3390/life13102071

**Published:** 2023-10-17

**Authors:** Pierpaolo Di Micco, Egidio Imbalzano, Giuseppe Camporese

**Affiliations:** 1 AFO Medicina, UOC Medicina Interna, P.O. Santa Maria delle Grazie, ASL NA2 Nord, 80078 Pozzuoli, Italy; 2 Department of Clinical and Experimental Medicine, University of Messina, 98122 Messina, Italy; egidio.imbalzano@unime.it; 3 General Medicine Unit, Thrombotic and Haemorrhagic Disorders Unit, Department of Internal Medicine, University Hospital of Padua, 35128 Padua, Italy; giuseppe.camporese@aopd.veneto.it

In recent years, great efforts have been made to improve decision making in caring for patients of venous thromboembolism (VTE). Indeed, the last decade has seen major advances in the development and management of new diagnostic and therapeutic protocols for the fight against VTE, a disease strongly associated with increased morbidity and mortality worldwide. Therefore, this Special Issue of *Life*, entitled “The new Frontier of Venous Thromboembolism”, publishes several contributions in this field. The focus of these articles primarily concerns therapeutic aspects of antithrombotic treatments for unprovoked VTE and cancer-associated thrombosis (CAT).

Although direct oral anticoagulants (DOACs) have been identified as the first choice of treatment for VTE, the presence of CAT complicates their application. Existing guidelines suggest different durations of therapy in these different clinical scenarios (i.e., for patients with CAT and without CAT). There are several registries worldwide that seek to better understand the long-term prognosis of CAT patients treated using DOACs. Such information is available in western countries in particular, and new data are emerging in this clinical setting. Tailored pharmacological prophylaxis and therapies are needed to treat specific clinical conditions, e.g., the long-term placement of central venous catheters to deliver adjuvant or neoadjuvant chemotherapy, as reported by Rydell et al. [1]. In addition, specific educational interventions are still needed, as reported by Zalunardo et al. [2], although the association between cancer and thrombosis is well known. Therapeutic updates on CAT have also been reported by Bernardi et al. using data from an Italian registry named MAC [3]. This report offers data about the extended phase of anticoagulation in patients with CAT, a topic that remains a matter of discussion in daily clinical practice [3].

Of course, other new insights into long-term treatment were also reported in the MAC project for patients with unprovoked VTE by Bortoluzzi et al. [4]. New insights into the long-term treatment of VTE using DOACs such as rivaroxaban have been reported in this Special Issue. Additionally, patients that took rivaroxaban monotherapy for VTE according to dose regimens suggested and advised against in international guidelines were discussed by Di Micco et al. [5]. Intriguing data were summarized in a review by Shah et al. [6] regarding catheter-directed DVT treatments, helping to address the contemporary lack of randomized clinical studies in the literature [6].

Another hot topic in the long-term treatment of VTE is related to the laboratory monitoring of anticoagulant activity. This practice can not only improve the safety of these treatments but is also useful when a sudden surgery is needed in patients using these drugs daily, as reported by Graf et al. [7]. This technique is always present in daily clinical practice and is used in particular in patients with a variable balance between the risk of recurrence and risk of bleeding. This report is important after more than 10 years of use of DOACs.

Therefore, the diagnosis and treatment of VTE is in continuous evolution and new insights are always welcome in order to improve outcomes and the quality of life of VTE patients. This Special Issue reports several hot topics, with a focus primarily on the long-term treatment of patients affected by VTE during oncological (or non-oncological) diseases. DOACs remain most commonly used for the treatment of VTE. However, their action is focused primarily on the final phases of clotting cascade. As such, their interaction with endothelial dysfunction is poor. Yet, endothelial dysfunction is one of more intriguing aspects of VTE pathogenesis, as reported by Chang JC [8], and it also represents a hot topic for future era of VTE.

We should therefore consider this SI to be a step towards the future evolution of this clinical picture. We are sure that readers will find this edition and future updates useful. 
